# Polar biodiversity data: From a national marine platform to a global data portal

**DOI:** 10.1016/j.patter.2022.100566

**Published:** 2022-08-30

**Authors:** Petra ten Hoopen, Helen J. Peat, Peter Ward, Geraint A. Tarling

**Affiliations:** 1 British Antarctic Survey, High Cross, Madingley Road, Cambridge CB3 OET, UK

**Keywords:** Antarctic, Arctic, biodiversity data workflow, data integration, oracle, GBIF

## Abstract

Global access to accurate biodiversity data is a prerequisite to our progress in understanding biodiversity dynamics in ecosystems and any changes that are occurring. Despite recent major advancements in sharing data on the world’s species, one of the remaining challenges relates to the mechanics of guiding data systematically from its provenance to end users. It can take considerable effort to orchestrate a successful sampling campaign, manage samples obtained in often extreme, remote conditions and to secure preservation of, and access to, the acquired data. Here, we briefly describe biodiversity data flow from a polar ship to a national data repository and onward to a global data portal. This paper highlights a few crucial points in this process, which aims to provide information systematically into the mosaic of our polar species biodiversity knowledge. This flexible workflow can be modified for other data types and adopted by other data repositories.

## Highlights

• A workflow for polar marine biodiversity data preservation was developed
• Crucial aspects of the data sharing process are discussed
• The workflow is demonstrated using a zooplankton abundance dataset
• The workflow can be adapted for multiple domains and by other data centers

## The bigger picture

Development of a workflow by the UK Polar Data Centre that summarizes internal infrastructure and external systems and standards required in order to collect polar marine biodiversity data and share them on global data portals in a standardized format. It highlights crucial aspects of the data preservation and sharing process and provides an overview of procedures applied. The workflow is explained using an example of a zooplankton abundance dataset. This is being adapted by the data repository for other data types and can be adopted by other data repositories.

## Teaser

A workflow for preservation and sharing of polar marine biodiversity data has been developed by the UK Polar Data Centre. Pivotal aspects of the data preservation process are identified and the workflow explained using one example dataset. The workflow enables sharing data in a standardized format with global data portals, and can be adapted to other domains and by other data repositories.

## Introduction

Sustainable management of natural resources has rightly become a priority in recent years and biodiversity knowledge plays an important role in that, as reflected, for example, in the United Nations Sustainable Development Goals.^1^ Ecosystem assessments, which can be used to generate indicators to advise decision-makers on impact of fisheries, tourism, or climate changes and biodiversity, are a fundamental aspect of ecosystem health.^2^ To understand biodiversity changes, patterns, and relationships within and between ecosystems require well-organized, integrated, and accessible biodiversity information. Initiatives such as the Global Biodiversity Information Facility^3^ (GBIF), Ocean Biodiversity Information System^4^ (OBIS), or SCAR Antarctic Biodiversity Portal^5^ are instrumental for integration of, and access to, biodiversity information. Sharing of biodiversity data is facilitated by developing (1) data sharing tools, such as the GBIF integrated Publishing Toolkit^6^ (IPT), (2) information standards,^7^ (3) a species register^8^ (WoRMS), (4) informatics fora, such as the Biodiversity Next,^9^ (5) knowledge alliances,^10^ or (6) libraries, such as the Biodiversity Community Integrated Knowledge Library.^11^ However, it is the responsibility of data contributors to provide validated and standardized data to the aggregator resources. While individual scientists can submit data to the global data portals, it is often an institutional data information facility or a data repository that enables access to the data on the data originator’s behalf.

Here, we describe how biodiversity information travels from its provenance of a marine platform in a polar ocean to a national data repository, the UK Polar Data Centre (UK PDC),^12^ and how it is managed at UK PDC and shared with the global user community. We identify crucial aspects of this journey and believe that this can offer guidance for others, as well as allowing us to improve the complex marine campaign workflows that have been established over decades by scientists, marine engineers, laboratory technicians, operation managers, data custodians, and informatics specialists at the British Antarctic Survey (BAS) during expeditions to Antarctic and Arctic seas.

## Results

**Figure 1:**
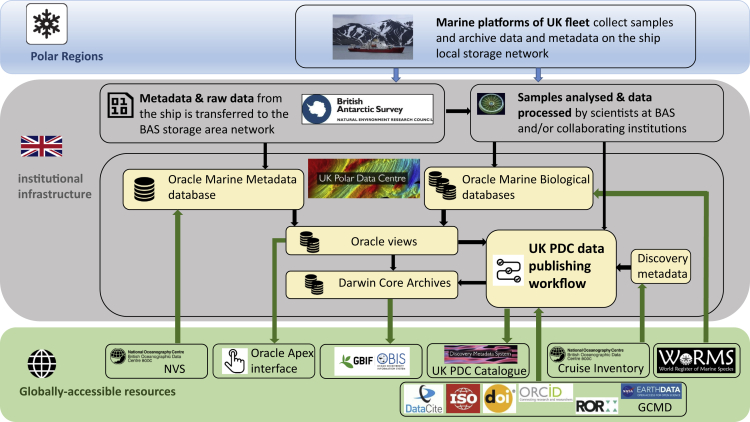
Polar marine data flow: Schematic representation showing the process of biodiversity information flow from its provenance in polar oceans to the UK Polar Data Centre and ultimately to the global user community NVS, NERC Vocabulary Server; WoRMS, World Register of Marine Species; DataCite, Metadata Schema of the DataCite registration agency, ISO 19115 Geographic Information Metadata standard of the International Organisation for Standards; ORCID, Open Researcher and Contributor ID system; DOI, Digital Object Identifier system; ROR, Research Organisation Registry; GCMD, Global Change Master Directory keyword list.

The whole process of polar marine data flow is schematically explained in Figure 1, which describes a data flow into, and within, the UK PDC and usage of global information resources and tools in the data publishing process, including the use of relevant standards and persistent identifiers, such as the DataCite Metadata Schema,^13^ Digital Object Identifier system^14^ or the Research Organisation Registry.^15^

As an example of how this biological data publishing pipeline has been adopted, we provide the BAS Bongo Database, which contains mesozooplankton biodiversity information from Bongo and mini-Bongo nets collected by the BAS in polar regions between 1996 and 2013. The BAS Bongo dataset^16^ is publicly available from the UK PDC, GBIF,^17^ and OBIS^18^ data portals and described in detail by Ward et al. (see the related paper in this issue of *Patterns*).^19^

### From a physical sample to raw data

A scientific polar ocean expedition is a complex endeavor that requires significant resources and years of forward planning to secure ship time, prepare instrumentation, and ensure that required expertise is available on board. This includes not only the relevant scientific expertise but also support of (1) marine engineers to deploy all scientific instrumentation with the assistance of ship officers and crew, (2) laboratory managers to ensure correct sample storage, shipping, and long-term sample preservation, (3) IT specialists to set up and maintain data storage systems, and (4) data custodians to organize sampling metadata and link them to the data such that it is visible and available to the research community and beyond.

A number of aspects affect the sampling success and preservation of the acquired data. Below, we highlight a few aspects that we identified as important for successful biodiversity data preservation:

• Drafting a data management plan before an expedition that outlines what datasets will be generated, who is responsible for generation of each dataset, and who will manage the data over the longer term.
• Involving relevant repositories from the start, i.e., from the expedition planning phase.
• Establishing efficient communication between the expedition science party, science technical support, and logistics operators throughout the whole expedition life time. This is essential especially for complex multidisciplinary campaigns, such as those collecting biological data along with biogeochemical, oceanographic, or bathymetric data.
• Establishing a physical sample collection system that enables sample provenance data traceability and flow from the moment of sample collection to its on-ship and off-ship storage and to a custodian of the taxonomic data generated from these samples.
• Establishing a data collection system that enables the recording of sample provenance data alongside environmental and taxonomic data from the first day of the expedition. Unlike in the field of physical oceanography, data-logging systems for biological sampling are not common. The BAS Antarctic Marine Engineering team has developed a custom-built net monitoring system that records sampling depth along with ambient temperature. The BAS IT team has developed a Digital Event Logging system that is widely used, especially by biologists, and which enables scientists to record details of each scientific deployment event along with continuous atmospheric and surface water measurements. These include wind speed and direction, sea surface temperature, and salinity, thus providing valuable contextual information of environmental conditions for each biological sample.
• Linking raw data from net haul catches, which are either sorted immediately on board or analyzed post-cruise, to the captured metadata in the Digital Event Logger.
• Backing up data daily and transferring to a secure network on the mainland.

The new UK polar research vessel, the RRS Sir David Attenborough (SDA), will address these aspects and include them in its operations.

### From raw data to a national database

Marine samples, usually but not limited to plankton or benthic samples, analyzed either directly in onboard experiments or post-expedition, generate derived data that can easily become disconnected from the acquired raw data and collected provenance data. This creates gaps in information required later for data discovery or data reuse. Therefore, it is crucial to maintain sampling provenance linked to the data.

**Figure 2:**
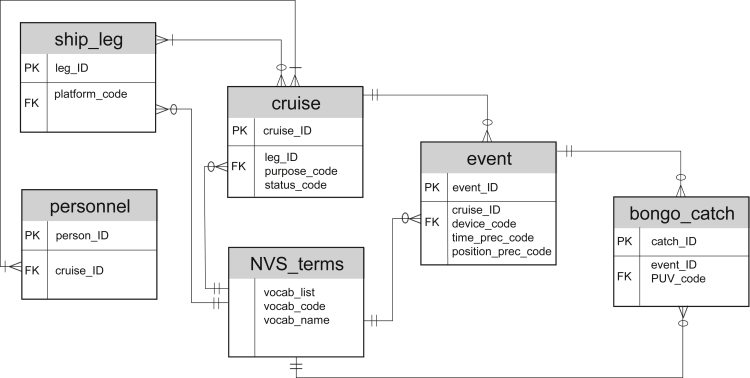
Entity relationship diagram schematically describing a logical framework of the database data structure Primary key (PK) and foreign keys (FK) are shown for each entity and cardinality relationships between entities are depicted using commonly used symbols for relationship types (mandatory, optional, one-to-one, and one-to-many).

The UK PDC maintains sampling provenance in a relational Marine Metadata Database of expeditions and deployment actions. Figure 2 depicts an entity relationship diagram of the database data structure. Each expedition, denoted often as a “cruise,” is described with its start and end dates, scientific focus, personnel, and ports of the ship leg. Each expedition comprises a series of scientific instrument deployments, denoted as “events,” each with a variety of descriptors, such as sampling device, deployment duration, depth, station designation, and geospatial coordinates. To ensure interoperability, the Marine Metadata Database uses the NERC Vocabulary Server^20^ (NVS) terminologies for description of device categories (L05), deployment devices (L22), and spatial and temporal precision (C07 and C28). The NVS, managed by the British Oceanographic Data Centre^21^ (BODC), consists of standardized and hierarchically organized vocabularies for indexing and annotating data and predominantly focuses on oceanography and related domains.

Expeditions represented in the database undertake on average 650 events, (ranging from 10 to over 6,100 events), where we record deployment details of each individual sensor. Currently we hold over 160,000 event records. This currently grows on average by over 4,000 events per year across all marine science disciplines. As research vessels grow in capability this is likely to increase due to longer expeditions with more sampling stations and more complex cruises with a greater variety of sensors. The workflow, however, does not have to change since this increase essentially means adding more records to the existing events schema or additional sensors using existing NVS terminologies. The relational database software is able to handle many millions of records. The Marine Metadata Database is thus storing essential expedition information that can be connected to other datasets containing either raw or processed data. For example, we hold also ship navigation data with over 6.7 million track points at 1 min resolution, with growth being on average 260,000 navigation data points per year. Connecting a database schema with one data type to another database of a different data type provides a flexible and scalable solution that can accommodate complex multidisciplinary data without duplicating deployment provenance information.

Management of biological data at UK PDC is explained below using the example of the BAS Bongo net dataset.^16^ For details of net sampling and analytical methodologies we would refer readers to the article by Ward et al.^19^

In brief, the whole plankton sample from each event is preserved in 10% (v/v) seawater/formaldehyde and labeled with provenance information, such as date, cruise, net type and mesh size, event, and station number, and whether the sample was entire or a known aliquot. Replicate samples are sometimes sorted to provide live plankton material for onboard experimentation.

Recording of sample information during collection is analog, usually on paper log sheets, but it is crucial that digitalization is carried out as soon as possible. Teams of scientists we are working with usually transfer data into prepared spreadsheet templates or a Microsoft Access database and these then serve as the master copy, although the paper log sheets are scanned and retained for cross-checking, if needed at a later stage.

After the cruise, in the home or a collaborating laboratory, samples are analyzed by splitting them into manageable aliquots and examined under a binocular microscope. Hundreds of individual species stages can be counted from each sample. Numbers are standardized to individuals per m^2^ through dividing by the estimated volume filtered (m^−3^) and multiplying by maximum sampling depth (m).

The taxonomic data and counts are handed over to the Data Centre. Data custodians verify scientific names from the taxon identifications against the WoRMS and map the data to the parameter codes of the BODC Parameter Usage Vocabulary^22^ (BODC PUV). Original names assigned on board are retained with no automated conversion to accepted names. Misspellings are identified using the WoRMS fussy search function and the data collector is consulted before any corrections are made. The taxonomic and biological data are collated into a biological Oracle database, where taxa and parameters measured are represented using the BODC PUV codes and linked to events within the Marine Metadata Database.

The standardized number of individuals for each taxon relate to each net sample of each cruise event. However, if sub-samples need to be created these can be handled in the database by maintaining two types of identifiers, one for the original sample created on the ship and another unique identifier for each sub-sample made from that original sample. The database can also hold data from any further analysis of the original sample, e.g., length measurements where the sample identifier is included, so linking the measurement made with all of the relevant collection information.

### From a national database to a global user

**Figure 3:**
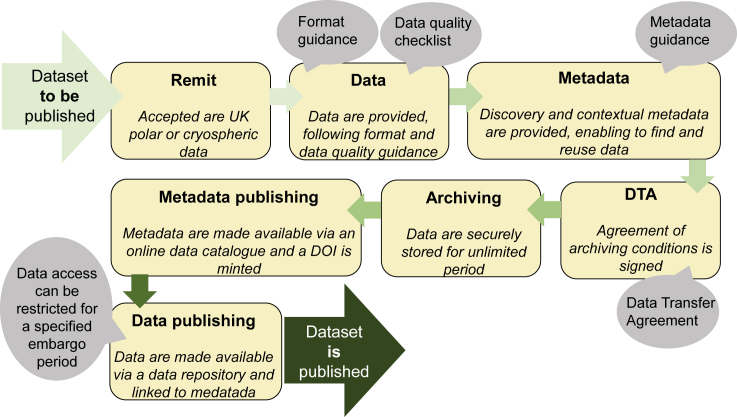
Data publishing workflow of the UK Polar Data Centre

Validated and standardized data archived in a relational database are safely preserved but can only be useful if made widely available. The UK PDC has established a data publishing workflow that was approved by the CoreTrustSeal^23^ certification and enables scientists to make their data available and discoverable via the online UK PDC metadata catalog and to receive accreditation via a formal data citation with a unique digital object identifier. The data publishing workflow is described at the UK PDC data deposit website and depicted in Figure 3. This workflow is used for any polar data of long-term value from UK-funded projects and is often triggered by a manuscript publication process. The manuscript-relevant data are available either directly as a set of files in an open data format or as a link to another resource, either internal, such as an Oracle database, or external, such as the BODC for oceanographic data or the EMBL-EBI^24^ for molecular data.

The UK PDC uses several ways to make polar biodiversity data available to global users. These are depicted in Figure 1 and detailed below using the example of the BAS Bongo Database.^16^

The biological Oracle database is linked to the Marine Metadata Oracle database into a materialized view. Discovery metadata are compiled primarily from cruise reports available from the BODC Cruise Inventory^25^ and together with the data in the materialized view made available via:

1. *A UK PDC discovery metadata catalog record*, which describes the data collection expeditions and methodology and provides a link to our data repository that holds the latest version of the data in the comma-separated value (csv) format, as shown in the example of the BAS Bongo dataset.
2. *An interactive online interface*, created using the Oracle APEX software, providing searchable access to the data within the materialized view. The interface enables users to search data per cruise, taxon, data type, geolocation, or date. The dataset can also be filtered for data from different depth ranges or from a specific month of the year. Moreover, the cruise identifiers can be expanded to view further details on each cruise and to access a full cruise report archived at the BODC Cruise Inventory. The whole database can be downloaded as a csv file directly from this interface. The interface also contains a map showing taxa distribution that has been created using a GeoServer service hosted by the BAS Mapping and GIS team. The map allows verification of the deployment data and shows data distribution, as seen in the interface example available from the BAS Bongo dataset metadata record.^16^
3. *An EML metadata record*. The biodiversity data together with the marine expedition metadata are converted into the Darwin Core format^26^ and then published using the integrated Publishing Toolkit.^6^ The BAS Bongo dataset, together with several other BAS datasets, has been published through the SCAR Antarctic Biodiversity Portal (biodiversity.aq), that feed both into GBIF and OBIS, using the biodiversity.aq IPT instance.^27^ The UK PDC is now setting up an IPT instance and will in the future be able to publish polar biodiversity datasets using the UK PDC IPT instance.^28^ The Oracle APEX interface, as described in the above point 2, contains the Darwin Core identifiers “EventID” and “OccurenceID,” which makes it possible to map each record from the APEX interface to the corresponding Darwin Core record in the GBIF/OBIS portal, should a user wish to do so.

## Discussion

Our experience shows that in order to enable safe preservation of biodiversity data and ensure they are findable, accessible, interoperable, and reusable (FAIR) it is good practice to:

• Establish a close collaboration between scientists and data management specialists throughout the whole data life cycle, in order to facilitate data processing and archiving and to ensure open access as soon as possible while respecting publishing rights of the data originator.
• Develop a sustainable, flexible, and scalable data management system that minimizes disconnection between data and metadata, and preserves sampling provenance.
• Create transparent and consistent workflows used by the data custodian to reduce and ideally remove any barriers in data flow from their provenance to end users.
• Adopt tools, best practice, domain-accepted terminologies, and persistent identifiers whenever possible in order to ensure interoperability with other data resources and to facilitate connection of an institutional data infrastructure with global data infrastructures.

The marine biological data workflow described here is transparent, consistent, scalable, and interoperable. It will be used by the Data Centre in the future for similar data types and can inspire other data repositories to build similar data publication pipelines to share data broadly following the FAIR data principles.

The polar marine biological data workflow can be used for marine species identification, e.g., pelagic or benthic organisms, human observations of cetaceans or birds, together with measurement of their characteristics, such as a weight, length, or body shape. While tabular biological data are usually not large in volume, typically in the range of kilobytes to megabytes, there is frequently several thousands of records per expedition. A long-term research program can generate a dataset in the range of a 100,000 records. For example, the Bongo net generated a dataset of 93,914 records over a period of 15 cruises. If a project generates images this increases the dataset volume to the range of gigabytes. The workflow we describe copes well with increasing volumes and types of data because: (1) relational databases are well suited to hold a large number of records, (2) new tables can be created for each new data type and linked to the existing biological database schema, (3) images can be stored on a server file system and linked to individual records of the relational biological database, and (4) many-to-one relationship exists (see Figure 2), where one event holds provenance metadata for many biological records originating from the same deployment. This means a biological record growth rate of ∼100% or greater per year can easily be accommodated.

A database schema can be created in a few weeks, data processing can be done within a few days to a few weeks, depending on the amount and quality of data handed over to the Data Centre and number of data validation consultations needed with the data originator.

Large datasets, such as the Bongo dataset, are usually compilations of extensive multi-partner programs or time series, and the time delay from the data collection to data sharing is dependent on the time researchers need to perform analyses on samples followed by quality control of the data before providing them to the Data Centre. This can range from a few weeks to a few years. However, once the relational database is designed for the program or sampling type, new data can be added at any point in time.

Optimalizations are being done on the database side to improve performance of data searches and visualization. For example, cruise tracks data are provided at 1 min resolution, rather than the resolution at which the data are captured to enable fast download and manageable volume. Relevant fields in the database tables are indexed to increase query speed.

The UK PDC uses the Marine Metadata Database not only in combination with biological databases but also with other types of data, such as a database of sediment sample characteristics and an environmental data portal for Tristan da Cunha, with oceanographic, imagery, and deck observation data. Expedition information is thus entered once and used multiple times. New data can be added at any time and, since the relational database tables or views are directly linked to the Oracle APEX application and the IPT, any changes can be promptly visualized for users. There are many relational database software options including open source ones such as Postgres. We have used Oracle, a commercial option as it also provides access to Application Express software, which makes it easy to create access to data held within the database using web pages.

A relational structure of provenance metadata, similar to the one illustrated in Figure 2, can also be used beyond the marine domain. For instance, in terrestrial field campaigns, where researchers take measurements and collect samples in a defined area, similar to deployment events undertaken from the ship. The recently added Event Core to the Darwin Core Standard^7^ to capture more complex biodiversity data beyond presence-only observations, suggests a broader applicability of the sampling event concept. Another example could be aerial surveys, where each campaign can be linked to a plane platform with mounted sensors collecting various data types during flights. It should be noted that interlinks to global data aggregators would be specific to the domain application.

Metrics of the data and usage statistics in terms of number of downloads are provided from the global data aggregators GBIF and OBIS. For example, the OBIS portal reports that the Bongo net dataset has appeared, in 2022, in 1,739 downloads (reported to date 13/06/2022).

Oracle APEX also provides metrics on page events with an average of 1,644 page events per month.

In the future, we would like to look into optimizing this workflow for better visualization of data in the relational databases using web mapping and development of migrations from Oracle databases to Postgres ones and vice versa. This will be needed for harmonization of the workflow with data streams from the RRS SDA, where Postgres is used for data acquisition.

Large compilations of biodiversity data preserved by the data center as described above receive good visibility and integration. However, smaller scale datasets that are published as supporting evidence of a scientific article are less well integrated. Sharing these individual datasets with GBIF in the Darwin Core format requires resources and close collaboration with the data originators and is one of the challenges we shall look into in the near future.

## Experimental procedures

### Resource availability

#### Lead contact

Further information and requests for resources and reagents should be directed to and will be fulfilled by the lead contact, Petra ten Hoopen (peopen@bas.ac.uk).

#### Materials availability

Samples referred to in this study have been deposited to the British Antarctic Survey Sample Stores and can be made available upon request.

## Data Availability

• Biological data from Bongo plankton samples have been deposited at the UK Polar Data Centre under https://doi.org/10.5285/5A711904-EF42-46A3-9F47-3F0D6B231F65 and are publicly available as of the date of publication. • This paper does not report original code. • Any additional information required to reanalyze the data reported in this paper is available from the lead contact upon request.
